# The impact of nano zinc on broiler productivity, tibia bone characteristics, and gut microbiota

**DOI:** 10.1038/s41598-025-18522-9

**Published:** 2025-09-30

**Authors:** Ahmed Samy, Mamdouh O. Abd-Elsamee, Suzan M. E. Ezzelarab, Hoda A. Kabary, Hany M. R. Elsherif

**Affiliations:** 1 Department of Animal Production, National Research Centre, Giza, 12622 Egypt; 2 Animal Production Department, Faculty of Agriculture, Cairo University, Giza, Egypt; 3 Agricultural Microbiology Department, National Research Centre, Cairo, 12622 Egypt

**Keywords:** Broiler, Nano zinc oxide, Productive performance, Intestinal microbiota, Tibia bone characteristics, Biotechnology, Microbiology, Zoology

## Abstract

This study aimed to investigate the impact of varying concentrations of nano zinc oxide compared to the conventional form affected the productivity, tibia bone characteristics, and cecal and intestinal microbiota in broilers from 1 to 35 days of age. The Arbor Acres 360-day-old male chicks were divided into six treatment groups (6 groups × 6 replicates × 10 chicks each). Except for zinc, which was given at three levels of 100% of the strain guide necessary level (110 mg), 50% (55 mg), and 25% (27.5 mg) of traditional or nano zinc oxide, six diets were formulated to meet the nutritional requirements of Arbor Acres chicks. The findings indicated that birds fed diets containing nano zinc oxide exhibited superior productive performance. There were no statistically significant differences (*P* > 0.05) observed between the various levels of nano zinc oxide. Furthermore, the study documented the ideal characteristics of the tibia bone in birds that were provided with diets supplemented with nano zinc oxide. These characteristics encompassed tibia weight, length, width, and breaking strength. Moreover, the birds that were fed nano zinc oxide had the best levels of tibia bone ash, calcium, phosphorus, and zinc content. The bone mineral density and concentration of birds fed nano zinc oxide were assessed using X-ray and dual-energy X-ray absorptiometry (DXA) techniques, revealing the most optimal results. The addition of zinc oxide in nano form led to enhanced intestinal microbiota by enhanced beneficial bacteria and decreased harmful bacteria. From the current results, the addition of nano zinc oxide at 25% recorded the best productive inclusion of nano zinc oxide provided the best productive performance, bone quality and gut microbiota (Lactobacilli, Enterobacteriaceae, and *Salmonella*).

## Introduction

Zinc is the second-most abundant trace mineral in the body after iron^1^. Zinc considered as a most important trace mineral due to its functions and biological activities in several physiological, digestive, and metabolic processes in broilers^2^. Moreover, it had a widely roles as co-enzymes for many metabolic enzymes, hormones functions (ex: Growth hormone) and antioxidants properties for scavenging free radicals^3,4^. In addition to this vital role in the bone growth, development, strength and regeneration^4^. Zinc promoting the calcium and phosphorus regulating hormones which leading to an increase in tibial calcium and phosphate^5^. Therefore, adding zinc to broiler diets in inorganic forms, which may be chelated by phytate and need adding a large amount of inorganic sources, resulted in financial loss and raised some environmental concerns^6^. To address this issue, zinc was bound to an organic source like an amino acid, increasing the bioavailability of the metal^7^. Recently, the best method for enhancing the bioavailability of minerals in broiler feeding was thought to be nanotechnology^2,8–10^.

The inorganic salts that are frequently added to animal feed have low bioavailability due to the presence of certain substances that might hinder the absorption of both micro- and macro elements^11^. As a result, their increased mineral excretion could endanger the ecosystem by contaminating the aquatic and soil environments^12^.

Applying the solutions that nanotechnology has to offer could help reduce this adverse environmental impact while also improving the productivity, health, and performance of poultry^10^. Nowadays, Nanotechnology is an emerging science field that have a hugely positive impact on all facets of daily life, including poultry nutrition^13^. Nanomaterial’s was very tiny in size less than 100 nm, so increasing the ratio of surface area to volume which gave the unique electrical, magnetic, optical, antibacterial and many other properties that will have enormous uses^14^. In broiler nutrition, the huge surface area leading to increasing the chemical reactivity, absorption and bioavailability of nanomaterials compared to conventional form^2^.

Due to its unique properties, zinc oxide nanoparticles are employed in a variety of applications, including antimicrobial, medication delivery, batteries, nutrition, and fertilizers. Additionally, it was approved by the USFDA as generally recognized as safe additives for use in food and feed^15^.

Supplementation of zinc oxide NPs for long term to broiler chicks has not been shown to have any negative effects on normal histology of the liver, blood physiology, immune system, and DNA damage of liver and bone marrow. These factors are crucial for validating ZnONPs for use in feed. ^16^.

Recently conducted studies have demonstrated that utilizing a little amount of nanomaterials was sufficient compared to using them in their usual form. Due to its size, availability, and absorption, as zinc in nano-form has a higher bioavailability than zinc from traditional sources (organic and inorganic)^17^. Also, Hussan et al.^18^ concluded that adding ZnONPs in broilers’ diets at a lower concentration than using a conventional source enhanced their body weight and feed efficiency. It was emerged that adding ZnONPs at half the recommended dosage produced performance results similar to those of traditional zinc without having any negative effects on broiler chickens^15,19^. ZnONPs supplementation to broiler diets at 40 or 60 mg/kg improved productive performance^20^.

At the required level, zinc is crucial for broiler chickens’ ideal bone development, growth, and strength^21,22^. Zinc plays a critical function in protein and collagen synthesis, which makes it necessary for healthy skeletal development and bone homeostasis^4^. Additionally, zinc at the right concentration increases hormones that control calcium and phosphate, increasing the amount of calcium and phosphate in the tibia^23^. Mohd Yusof et al.^22^ found that supplementing with ZnONPs at low levels had a similar impact on tibia bone characteristics (tibia bone weight, length, thickness, and mineralization) as adding with the recommended amount of zinc to the broiler diet. In the same line, Samy et al.^2^ concluded that supplementing broiler diets with ZnONPs from 1 to 35 days of age gave the best results for weight gain and feed conversion ratio, as well as improved tibia bone characteristics (tibia bone length, width, breaking strength, ash, mineral density, and mineral concentration) without having any adverse effects on thyroid hormones or kidney and liver functions.

Therefore, this study aimed to investigate the impact of zinc oxide nanoparticles on the productive performance, tibia bone characteristics and gut microbiota.

## Materials and methods

### Ethics approve

This study received ethical approval (Approval No. ARC-APRI-25–24) from the Institutional Animal Care and Use Committee (ARC-IACUC), Agricultural Research Center. The study was conducted in accordance with the ARRIVE guidelines (https://arriveguidelines.org). All experimental procedures were performed in accordance with relevant institutional and national regulations.

### Anesthesia and euthanasia declaration

All animal-related treatments were carried out in compliance with accepted veterinary standards. To provide sufficient sedation and pain relief, a combination of xylazine (2–4 mg/kg) and ketamine (20–40 mg/kg) was used to induce anesthesia. According to the American Veterinary Medical Association’s (AVMA) Guidelines for the Euthanasia of Animals (2020), 80% CO₂ was inhaled and then progressively delivered into the chamber at a displacement rate of roughly 20–30% of the chamber volume per minute. By causing hypoxia, which results in unconsciousness and eventual euthanasia, this technique guarantees a compassionate and painless death.

### The experiment site

The Poultry Nutrition Research Unit (PNRU), located at Cairo University’s Agriculture College in Giza, Egypt, was the place of the field experiment. All laboratory analyses were also carried out at Egypt’s National Research Centre.

### Experimental materials

A total of 360 one day-old male broiler chicks (Arbor Acres strain) were obtained from a local commercial hatchery and used as the animal model in this study.

Zinc oxide (ZnO) was purchased from a chemical supplier, while nano zinc oxide (nZnO) was synthesized chemically using the Sol–Gel technique, as described in previous studies^13,14,24^.

### Experimental design and diets

The chick growth experiment was conducted to investigate the impact of zinc oxide nanoparticles on broiler productive performance and bone characteristics from 1 to 35 days of age. In this study birds were raised in pens where 360 one day-old male chicks (Arbor Acres) were assigned into six treatment groups (6 groups × 6 replicates × 10 chicks each). Six diets were prepared to cover all the nutrient needs of Arbor Acres chicks except zinc, which was added, in 2 sources × 3 levels, at 100% (of the recommended level according to the strain guide), 50%, and 25% of traditional or nano zinc oxide, corresponding to 110 mg, 55 mg, and 27.5 mg, respectively. Table 1 shows the composition of all experimental diets throughout various periods.

**Table 1:** Formulation and nutrient composition of starter, grower and finisher diets.

	Starter	Grower	Finisher
Yellow corn	51.82	56.58	61.88
Soybean meal (44%)	36.2	29	25.2
Corn Gluten meal (60%)	5	6.14	5
Soybean oil	3	4.2	4.5
Dicalcium phosphate	1.3	1.5	1.3
Limestone	1.6	1.6	1.3
Vit and Min Mix^a^	0.3	0.3	0.3
NaCl	0.3	0.3	0.3
L-lysine HCl	0.28	0.26	0.17
Dl-methionine	0.13	0.08	0.05
Therionine	0.07	0.04	0
Total	100	100	100
Calculated composition%^b^			
Crude protein	23.02	21.03	19.08
ME (Kcal/Kg)	3007	3159	3237
Lysine%	1.44	1.25	1.06
Methionine%	0.52	0.45	0.39
Methionine + cystine%	0.91	0.81	0.72
Therionine%	0.94	0.83	0.71
Tryptophane%	0.32	0.27	0.24
Valine%	1.1	1	0.91
Calcium%	1.01	1.03	0.87
Nonphytate P%	0.53	0.52	0.46

^a^Vitamin–mineral mixture supplied per Kg of diet: Vit A, 12,000 IU; Vit D3 , 2200 IU; Vit E, 10 mg; Vit K3 , 2 mg; Vit B1 , 1 mg; Vit B2 , 4 mg; Vit B6 , 1.5 mg; Vit B12, 10 g; Niacin, 20 mg; Pantothenic acid, 10 mg; Folic acid, 1 mg; Biotin, 50 g; Choline chloride, 500 mg; Copper, 10 mg; Iodine, 1 mg; Iron, 30 mg; Manganese, 55 mg; and Selenium, 0.1 mg.

^b^According to NRC 1994.

The initial weights of chicks in all replicates were close to each other about 42 g. Chicks were raised in a warm environment and fed the experimental diets during starter (1- 10d), grower (11-25d) and finisher (26-35d) periods. The chicks were given unlimited access to water and feed. Throughout the experimental trial, light was provided for 16 h/day.

### Measured parameters

#### Productive performance

The chicks were weighed after an overnight fast, and the amount of feed consumed each week by each repeat was noted. Body weight gain (BWG) and the feed conversion ratio (FCR) were calculated.

#### Slaughter and carcass characteristics

At 35 days of age, six chicks per treatment were slaughtered in order to assess the intestinal and cecal microbiota, and the features of the tibia bone. Reduce bird stress during travel and handling, then relax them before slaughter. The bleeding process should be quick and thorough to ensure rapid death, this is done while they are unconscious to prevent pain.

#### Tibia bone characteristics

Tibia bone characteristics were conducted in right tibia bone, which removed from the six slaughtered chicks per each treatment. Preparation of each tibia bone by removed adhering flesh and lipids using diethyl ether. Tibia bone weight, length and width were measured after that dried in oven for 3 h at 105 °C.

Digital Force Gauge apparatus was used for measured the tibia bones breaking strength according to Mašić^25^.

Dried tibia samples were ashed in a muffle furnace at 600 °C for 6 h, and the tibia ash content was calculated as a percentage of the tibia dry weight. The concentrations of calcium, phosphorus, and zinc in the tibia ash were determined following the dry digestion method described in the Official Methods of Analysis^26^. Subsequently, Ca, Zn, and P levels were measured using commercial diagnostic kits (Química Clinica Aplicada S.A., Spain).

Using X-ray equipment, radiographs were obtained to determine how different treatments affected the mineral concentration of the tibia bones^9^.

Dualenergy X-ray absorptiometry (DXA) was used to measure the tibia bone densitometric analysis of the Bone mineral density (BMD) and bone mineral content (BMC), Dedicated to small animals, the Norland Excell Plus Densitometer (Norland, Fort Atkinson, WI, USA) is outfitted with Norland Illuminatus small Subject Scan software version 4.3.1^27^. The measures for bone mineral density and concentration are given in grams per cubic centimeter and grams, respectively.

#### Characterization and enumeration of intestinal and cecal microbiota

Additionally, duodenal and cecal microbes were enumerated to detect the effect of the zinc oxide nanoparticles compared to the conventional source, on specific types of intestinal bacteria (Aerobic, Anaerobic, *Lactobacilli*, *Enterobacteriaceae*, and *Salmonella*). The intestinal and cecum parts were removed in sterilized petri dishes from the slaughtered birds. Under aseptic condition, 1 g of duodenum and cecum digesta was transferred directly to clean test tubes contains 9 ml of sterilized saline. A tenfold dilution was prepared from the digestion content of each part, followed by direct seeding of 1 ml of each dilution on the pre-warm selective media^28–30^. Nutrient agar medium was used for determination of total aerobic and anaerobic bacterial counts, and the plates were incubated either aerobically or anaerobically in McIntosh jars with a burning cotton piece (until the complete exhaustion of the atmospheric air), at 30°C for 24–48 h. MacConkey agar was used for total Enteric bacteria count^31,32^. The cultures were left to grow aerobically at 37 °C for 48 h. *Lactobacilli* count was detected by inoculating in MRS medium^33^, followed by incubation at 37 °C for 48 to 72 h. Finally, *Salmonella* count was estimated by inoculating on SS (Salmonella, Shigella) agar plates^32,34^, and incubated for 48 h at 37°C.

### Statistical analysis

The General Liner Model of SAS^35^ was used to statistically evaluate the data using two-way analysis of variance. Duncan’s new multiple range test^36^ was used to detect the difference between among means at (*P* < 0.05). The model was Y_ijk_ = μ + Z_i_ + L_j_ + ZL_ij_ + E_ijk_.

Where Y_ijk_ is the observation of the parameter measured, μ is the overall mean; Z_i_; is the fixed effect of the zinc source, L_j_ is the fixed effect of the zinc level, ZL_ij_ is the effect of the interaction between source and level, and E_ijk_ is random error.

## Results

### Productive performance

Tables 2 and 3 showed the effect of zinc on broiler productive performance during starter, grower, finisher and overall periods.

**Table 2:** Effect of dietary treatments on growth performance of broiler during starter and grower periods.

Item		Starter (1–10 days)			Grower (11–25 days)		
		BWG	FI	FCR	BWG	FI	FCR
Zinc oxide level %	Zinc oxide source						
100%	CZnO	249^a^	341	1.37^b^	677^b^	1000	1.48
50%	CZnO	189^b^	320	1.69^a^	641^b^	920	1.44
25%	CZnO	178^b^	315	1.77^a^	609^b^	1009	1.66
100%	NZnO	280^a^	302	1.07^c^	785^a^	1146	1.46
50%	NZnO	284^a^	302	1.06^c^	789^a^	1140	1.44
25%	NZnO	257^a^	297	1.16^c^	759^a^	1097	1.45
SE of means		± 13.37	± 43.33	± 0.06	± 22.07	± 99.82	± 0.13
Main effects							
Source							
CZnO		205^b^	325	1.61^a^	642^b^	976	1.53
NZnO		274^a^	300	1.10^b^	778^a^	1128	1.45
SE of means		± 7.72	± 25.02	± 0.08	± 23.11	± 57.63	± 0.073
Level %							
100%		264	322	1.22	731	1073	1.47
50%		236	311	1.38	715	1030	1.44
25%		218	306	1.47	684	1053	1.56
SE of means		± 19.45	± 30.64	± 0.30	± 19.59	± 70.58	± 0.09
Significances							
Source of variation							
Source effect		***	NS	***	***	NS	NS
Level effect		NS	NS	NS	NS	NS	NS
Source × Level		**	NS	*	***	NS	NS

Means designated with the same letter in the same column are not significantly different at 0.05 level of probability.

**Table 3:** Effect of dietary treatments on growth performance of broiler during grower and overall periods.

Item		Finisher (26–35 days)			Overall (1–35 days)		
		BWG	FI	FCR	BWG	FI	FCR
Zinc oxide level %	Zinc oxide source						
100%	CZnO	824ab	1333	1.62b	1750b	2675	1.53bc
50%	CZnO	764bc	1324	1.73b	1584c	2564	1.62b
25%	CZnO	748c	1389	1.86a	1545c	2713	1.76a
100%	NZnO	864a	1390	1.61b	1929a	2838	1.47c
50%	NZnO	902a	1491	1.65b	1975a	2933	1.48c
25%	NZnO	854ab	1393	1.63b	1870a	2787	1.49c
SE of means		± 32.91	± 100.01	± 0.04	± 33.90	± 192.72	± 0.03
Main effects							
Source							
CZnO		779b	1349	1.74	1626b	2650	1.64a
NZnO		873^a^	1425	1.63	1924a	2853	1.48b
SE of means		± 20.73	± 57.74	± 0.08	± 25.13	± 111.26	± 0.05
Level %							
100%		844	1362	1.61	1839	2756	1.5
50%		833	1408	1.69	1779	2748	1.54
25%		801	1391	1.74	1707	2750	1.62
SE of means		± 25.39	± 70.72	± 0.09	± 61.48	± 136.27	± 0.06
Significances							
Source of variation							
Source effect		***	NS	NS	***	NS	***
Level effect		NS	NS	NS	NS	NS	NS
Source × level		*	NS	*	***	NS	***

Means designated with the same letter in the same column are not significantly different at 0.05 level of probability.

The results showed no significant differences (*P* > 0.05) were recorded among all treatments in main source, level or interaction (source × level) of feed intake. Which indicated that the addition of nano or traditional source of zinc at different levels had not affect the feed intake of broiler chicks at different periods.

In source main effect, the addition of nano zinc significantly enhanced (*P* ≤ 0.05) body weight gain (BWG) in all periods compared to the traditional source. Also, in source main effect the addition of nano zinc significantly improved (*P* ≤ 0.05) FCR in the starter and overall periods while no significant (*P* > 0.05) differences recorded in grower and finisher periods.

In level main effect of BWG and FCR no significant (*P* > 0.05) differences recorded in all periods.

In interaction (source × level) the addition of nano zinc at different levels gave the best (*P* ≤ 0.05) BWG in all periods. In starter and overall period, the worst BWG and FCR recorded for birds fed 50% and 25% of traditional zinc. In finisher period, the worst BWG and FCR recorded for birds fed 25% of traditional zinc.

### Tibia bone measurements

Table 4 shows the main effect of source, level and the interaction (source × level) of zinc on the bone characterization.

**Table 4:** Effect of dietary treatments on tibia bone characterization of broiler.

Item		Weight (g)	Length (mm)	Width (mm)	Breaking strength (Kgf)
Zinc oxide level %	Zinc oxide Source				
100%	CZnO	7.88b	87.72b	7.44b	8.44b
50%	CZnO	7.14b	86.73b	6.99b	5.99b
25%	CZnO	6.82b	80.49c	7.25b	5.47b
100%	NZnO	12.24a	95.38a	9.12a	16.09a
50%	NZnO	13.09a	96.12a	9.53a	15.01a
25%	NZnO	12.20a	97.70a	9.73a	14.34a
SE of means		± 0.37	± 1.99	± 0.28	± 0.95
Main effects					
Source					
CZnO		7.28b	84.98b	7.23b	6.63b
NZnO		12.51a	96.40a	9.46a	15.15a
SE of means		± 0.22	± 1.15	± 0.16	± 0.55
Level %					
100%		10.06	91.55	8.28	12.26
50%		10.12	91.42	8.26	10.5
25%		9.51	89.09	8.49	9.91
SE of means		± 0.26	± 1.41	± 0.19	± 0.67
Significances					
Source of variation					
Source effect		***	***	***	***
Level effect		NS	NS	NS	NS
Source × level		***	***	***	***

Means designated with the same letter in the same column are not significantly different at 0.05 level of probability.

Tibia bone weight had significantly (*P* ≤ 0.05) improved by the addition of the nano zinc (12.51 g) compared to the traditional source (7.28 g). Whereas, in the interaction (Source × level) the treatments of nano zinc significantly increased (*P* ≤ 0.05) the weight of tibia bone at 100, 50, 25% by 12.24, 13.09 and 12.20 g, respectively compared to the traditional source at the same levels about 7.88, 7.14, 6.82 g, respectively.

Tibia bone length had significantly (*P* < 0.05) enhanced by the addition of nano zinc (96.40 mm) compared to the traditional source (84.98 mm). The addition of nano zinc at 100, 50 and 25% improved tibia bone length by 95.38, 96.12 and 97.70 mm, respectively compared to traditional source at the same levels 87.72, 86.73 and 80.94, respectively. In the source effect on the tibia bone width, the addition of nano zinc significantly (*P* ≤ 0.05) improved by 9.46 mm compared to the traditional one by 7.23 mm.

In the interaction (source × level), the addition of nano zinc 100, 50, 25% increased the tibia bone width by 9.12, 9.53 and 9.73 mm, respectively compared to the same levels of the traditional levels about 7.44, 6.99 and 7.25 mm, respectively. The tibia bone breaking strength had the same trend of the tibia weight and width. Which the addition of nano zinc improved the tibia breaking strength by 15.15 Kgf compared to traditional source 6.63 Kgf. Also, in the interaction effect (source × level), the addition of nano zinc by 100, 50 and 25% enhanced the breaking strength by 16.09, 15.01 and 14.34 Kgf, respectively compared to the traditional source at the same levels by 8.44, 5.99 and 5.47 Kgf, respectively.

While the main effect of level had no significant differences (*P* > 0.05) in tibia bone weight, length, width and breaking strength.

The percentage of tibia ash, in addition to the amounts of calcium (Ca), phosphorus (P), and zinc (Zn), are shown in Table 5.

**Table 5:** Effect of dietary treatments on tibia bone ash and minerals.

Item		Ash (%)	Calcium (mg/dl)	Phosphorus (mg/dl)	Zinc (µg/dl)
Zinc oxide level %	Zinc oxide source				
100%	CZnO	46.67ab	37.42b	20.60b	236b
50%	CZnO	43.57bc	35.92b	18.49c	227bc
25%	CZnO	42.33c	34.88b	17.85c	216c
100%	NZnO	50.01a	43.89a	22.83a	254a
50%	NZnO	50.33a	42.66a	22.43a	250a
25%	NZnO	49.67a	42.64a	22.09a	250a
SE of means		± 1.22	± 1.50	± 0.28	± 5.01
Main effects					
Source					
CZnO		44.25b	37.25b	19.13b	227b
NZnO		49.40a	41.40a	21.90a	248a
SE of means		± 0.88	± 1.37	± 0.51	± 4.18
Level %					
100%		48.33	40.67	21.67	245
50%		47	39.33	20.5	238
25%		46.01	38.67	19.83	233
SE of means		± 1.07	± 1.65	± 0.62	± 5.04
Significances					
Source of variation					
Source effect		***	***	***	***
Level effect		NS	NS	NS	NS
Source × level		***	***	***	***

Means designated with the same letter in the same column are not significantly different at 0.05 level of probability.

In the main effect of level, no significant differences (*P* > 0.05) obtained in ash percentage, calcium (Ca), Phosphorus (P) and zinc (Zn) concentrations in broiler tibia bone.

In source main effect, the addition of nano zinc significantly enhanced (*P* ≤ 0.05) Ash %, Ca, P and Zn compared to the birds fed on the traditional source.

In the interaction (source × level) the best results (*P* ≤ 0.05) recorded for birds treated with nano zinc regardless the level of addition.

**Fig. 1:**
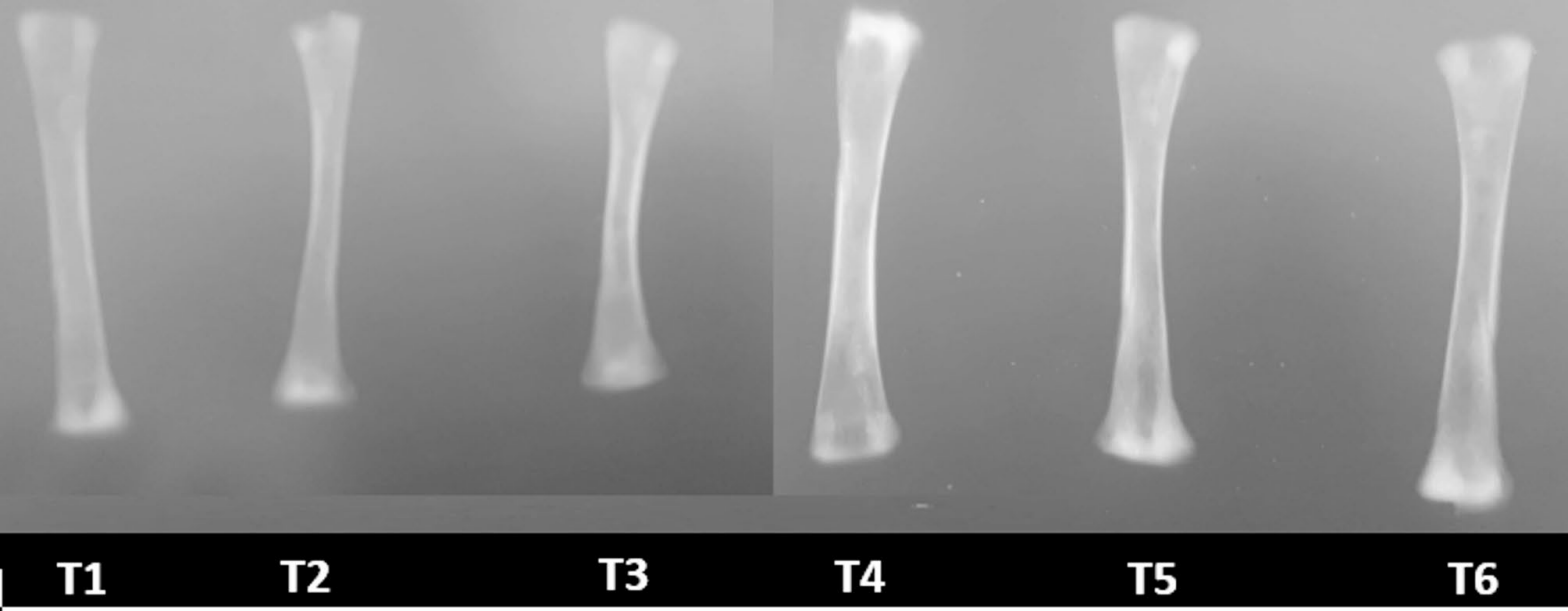
Radiographic image of the effect of different treatments on tibia bone scans using X-ray at 35 days of age.

**Fig. 2:**
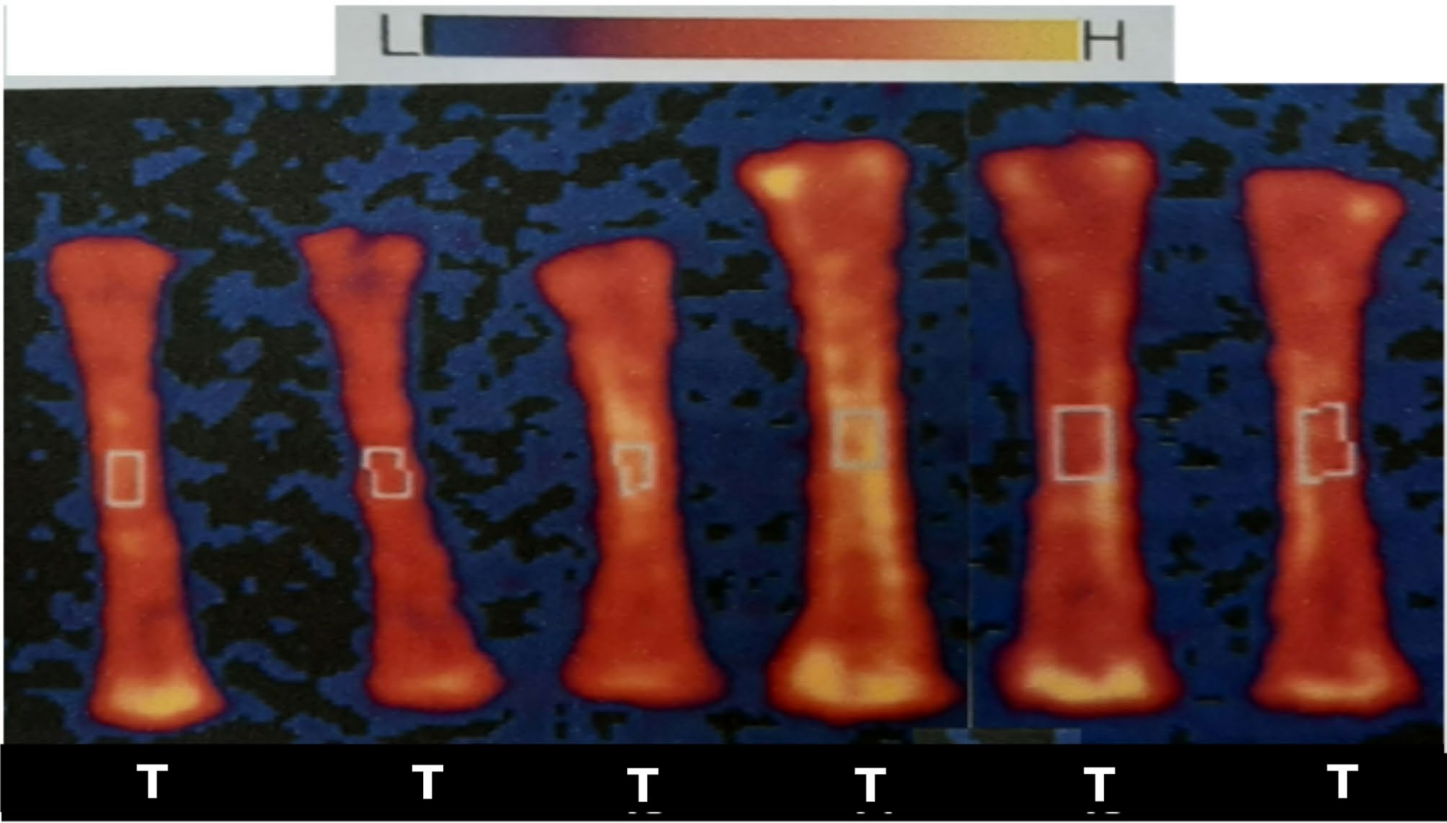
Radiographic image of the effect of different treatments on tibia bone scans using dual energy X-ray absorptiometry at 35 days of age.

From X-ray and DXA analysis of broiler tibia bone (Figs. 1, 2), the effect of bone mineral concentration (BMC) and bone mineral density (BMD) are shown in Table 6.

**Table 6:** Effect of dietary treatments on bone mineral density and concentration of broiler tibia bone.

Item		BMD (g/cm^3^)	BMC (g)
Zinc oxide level %	Zinc oxide source		
100%	CZnO	0.17^c^	0.08c
50%	CZnO	0.15d	0.05d
25%	CZnO	0.15d	0.05d
100%	NZnO	0.24a	0.20a
50%	NZnO	0.23b	0.19ab
25%	NZnO	0.23b	0.18b
SE of means		± 0.004	± 0.006
Main effects			
Source			
CZnO		0.16b	0.06b
NZnO		0.23a	0.19a
SE of means		± 0.002	± 0.003
Level %			
100%		0.21a	0.14a
50%		0.19b	0.12b
25%		0.19b	0.12b
SE of means		± 0.003	± 0.004
Significances			
Source of variation			
Source effect		***	***
Level effect		***	***
Source × level		***	***

Means designated with the same letter in the same column are not significantly different at 0.05 level of probability.

In the main source effect, the addition of nano zinc oxide significantly improved (*P* ≤ 0.05) the BMD and BMC by 0.23 g/cm^3^ and 0.19 g, respectively than the traditional zinc 0.16 g/cm^3^ and 0.06 g, respectively.

In the main level effect, the best level (*P* ≤ 0.05) recorded at level 100% of zinc regardless the source used for BMD and BMC which recorded 0.21 g/cm^3^ and 0.14 g, respectively.

In the interaction source × Level, the best (*P* ≤ 0.05) BMD and BMC recorded for 100% nano zinc by 0.24 g/cm^3^ and 0.20 g, respectively. Also, the addition of nano zinc at different levels had a superior effect on BMD and BMC. While the worst (*P* ≤ 0.05) BMD and BMC recorded for 50 and 25% traditional zinc which gave the same number 0.15 g/cm^3^ and 0.05 g, respectively.

### Gut microiota

**Fig. 3:**
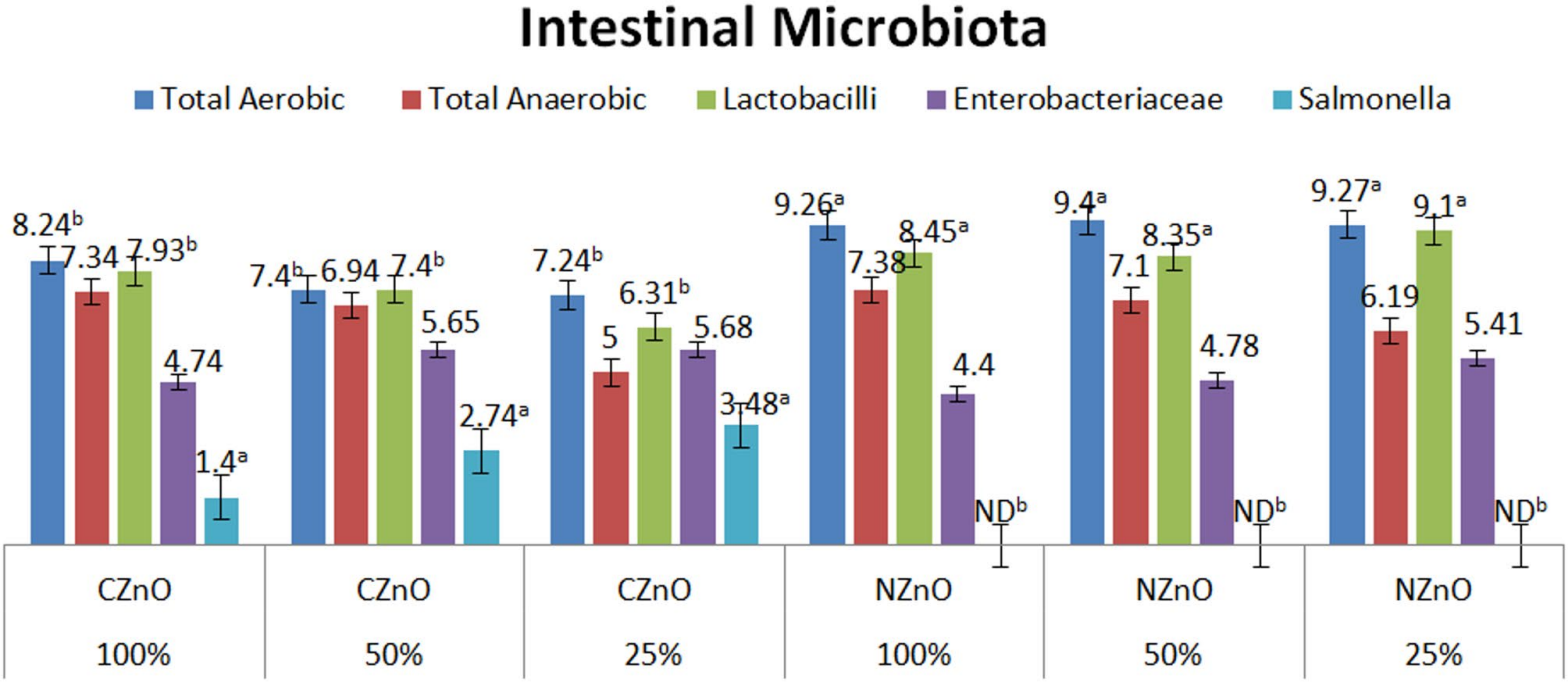
The effect of different treatments on the presence of selective types of duodenal bacteria (Log cfu).

The results of evaluating the effect of zinc oxide nanoparticles (ZnONPs) on duodenal bacterial count, in three different levels, are illustrated in Fig. 3. Overall, the addition of zinc nanoparticles to the chicken’s diet enhances the growth of beneficial bacteria with the restricting the growth of harmful intestinal types, compared to the birds fed on the conventional zinc. However, among the conventional zinc trials, the best intestinal bacterial results, detected when zinc concentration was 100 mg/kg. Also, the total aerobic, and total lactobacilli count, were significantly (*P* < 0.05) increased when the diet is supplemented with nano-zinc with favored concentrations at 50 and 25 mg/kg, and the best detection of lactic acid bacilli (log cfu = 9.1), when the diet contains 25 mg/kg. The existence of enteric bacteria was decreased when treating the diet with nano-zinc, where the log value of the bacteria colony forming unit cfu reached 4.4. Notably, the addition of zinc nanoparticles resulted in diminishing the presence of *Salmonella* bacteria to undetectable values for all trials, and all used concentrations.

**Fig. 4:**
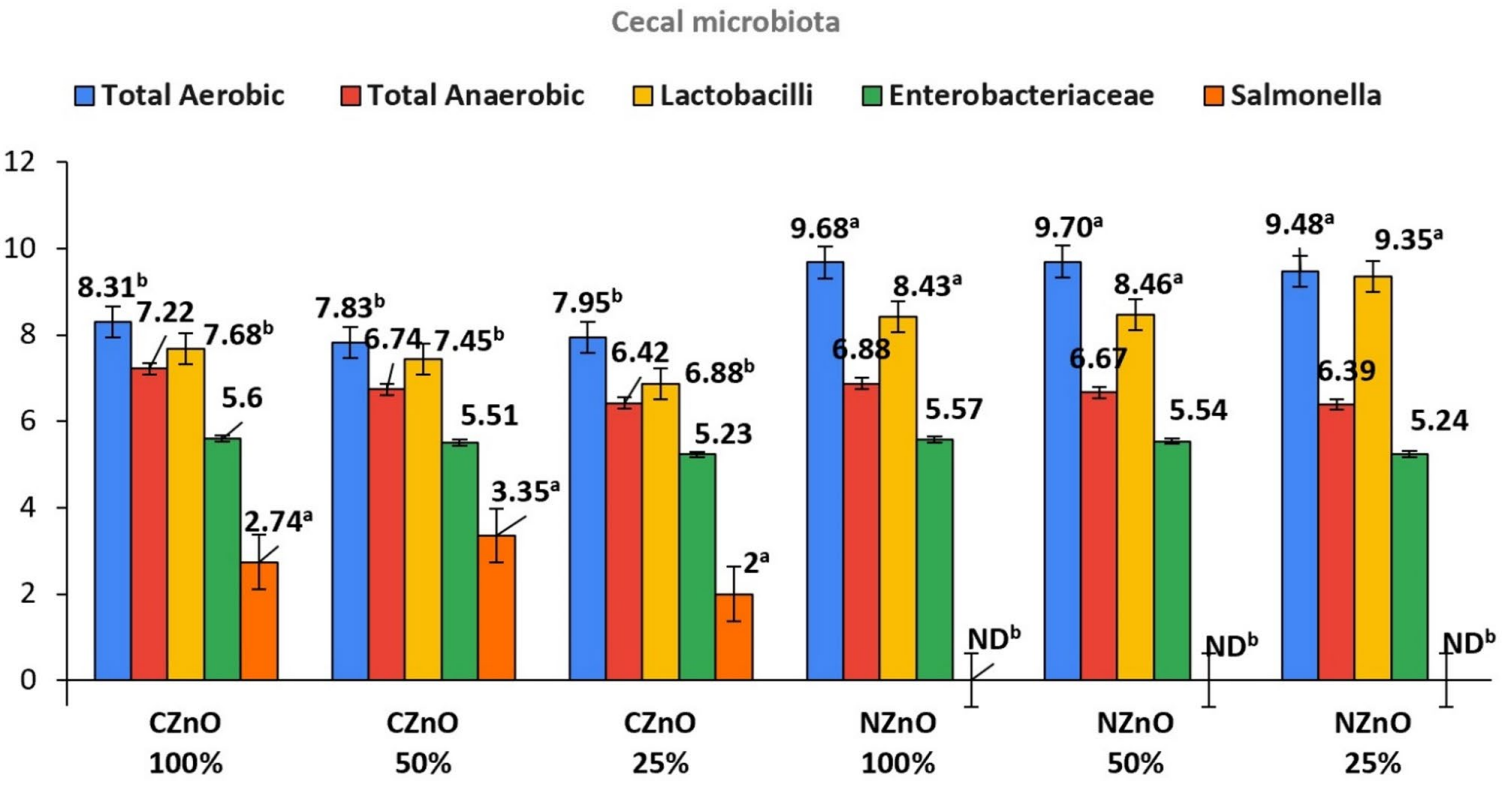
The effect of different treatments on the presence of selective types of cecal bacteria (Log cfu).

Correspondingly, the results of the cecal bacterial count exhibit a similar pattern to the duodenal bacteria count, when subjected to the two different zinc sources and their concentrations as shown in Fig. 4. Worthy to mention that the cecal bacterial counts is in general, greater than the duodenal bacterial count which may be related to the growth conditions and the type of digesta preferred to different bacterial populations, according to the exact site along the intestinal tract. Also, the study results illustrated that the same level of zinc nanoparticles (25 mg/kg) was the best in increasing the count of lactobacilli bacteria (log cfu = 9.35) and decreasing the count of Enterobacteriaceae (log cfu = 5.24). Alike the duodenal microbial analysis, *Salmonella* bacterial count wasn’t detected in the collected cecal samples in all zinc nanoparticles trials which support the previous experimental findings. Similar to the duodenal bacteria count obtained from the zinc conventional diet trial, the best bacterial counts obtained was detected when zinc concentration was 100% of recommended level (Lactobacilli count = 7.68 log cfu) which still significantly less than the count obtained for the same bacteria when zinc nanoparticles concentrations was 25% from recommended level.

## Discussion

The results of performance indicated that the addition of nano zinc to the broilers diets enhanced the BWG and FCR compared to the traditional form. These results are supported by many studies; the bioavailability of zinc in nano-form is superior to conventional zinc (organic and inorganic) sources, which could be attributed to its size, availability, and absorption^17^. The addition of ZnONPs to broiler diets from 1 to 35 days of age gave the best results (*P* < 0.01) in weight gain and feed conversion ratio without any adverse effect on thyroid hormones, liver and kidney functions^2,37^. Hussan et al.^18^ concluded that supplementation of ZnONPs at lower concentration than conventional source improved the body weight and feed efficiency of broilers. Zhang et al.^15^ found that the addition of ZnONPs at 50% from recommended level (40 mg/Kg) gave the comparable performance results to traditional zinc (80 mg/Kg). Kumar et al.^19^ found that dietary supplementation of hot-melt extruded zinc at the level of 55 mg/kg has the potential to be used as an alternative to conventional ZnSO4 at 110 mg/kg (ZnS) without any adverse effect on growth performance of broiler chickens. Eskandani et al.^38^ found that supplementation of ZnONPs at 30 mg/Kg to the broiler diets (1–42 days of age) enhanced growth performance, improved humoral immunity, Zn content of breast meat, and meat quality, while decreasing MDA content of breast meat. Hatab et al.^20^ concluded that supplementation of ZnONPs to broiler diet at 40 or 60 mg/kg improved productive performance.

Zinc reaching the optimal level in promoting calcium and phosphate-regulating hormones, which lead to increase in tibia Ca, and P^23^. Nano zinc sources are less reactive than inorganic forms of zinc causing more bioavailability and saving of zinc for collagen synthesis and osteoblast growth, it may explain the improvement of tibia breaking strength with nano zinc groups^21^. Samy et al.^2^ found that addition of ZnONPs to broiler diets from 1 to 35 days of age enhanced tibia bone characteristics (tibia bone length, width, breaking strength, ash, mineral density and mineral concentration). Mohd Yusof et al.^22^ found that supplementation of ZnONPs at low level had the comparable effect as supplementing recommended level of zinc to the broiler diet on tibia bone characteristics (tibia bone weight, length, thickness and mineralization) also enhanced zinc utilization. Zinc oxide NPs in the diet improved Zn absorption in broiler chickens. Additionally, tibia ash, Zn, Ca, and P retention showed enhanced tibia bone mineralization in broilers given 100 mg/kg of ZnONPs compared to the control^5^.

The obtained results were in correspondence with Yusof et al.^5^ who concluded that, the applications of zinc oxide nanoparticles in concentrations 70 mg/kg, resulted in improving lactic acid bacteria existence (log cfu = 6.60) and restricting the presence of Salmonella (log cfu = 0.43) in intestine, when compared to birds fed diets containing 100 mg/kg zinc oxide, which were 5.97 and 1.36, respectively. Simialry, Opoola et al.^39^ estimated the effect of applying 120 mg ZnO nanoparticles per kg diet on the intestinal lactobacilli count to be increased to 14.98 log cfu, in comparison to 12.88 log cfu for the basal diet.

It’s well known that the general body health is strongly correlated with an efficient gastrointestinal system, including the presence of beneficial GIT microbiota. Besides, the digestive role of these intestinal bacteria, the increase in the beneficial bacteria in intestine helps in improving the general immunity, regulating body hormones, and competing with other harmful bacterial types existing in the same intestinal environment^40,41^. Accordingly, Yusof et al.^5^ exhibited lactic acid bacteria count in chicken diet supplemented with ZnO nanoparticles (70 mg/kg) was 7.23 log cfu, which significantly better than the control diet results (6.33 log cfu). Alternatively, Opoola et al.^38^ estimated the lactic acid bacterial count in the cecum of broilers chicken after feeding on basal diet with 120 mg/kg ZnO nanoparticles to be 3.90 log cfu, compared to 2.00 log cfu for basal diet with no ZnO. Moreover, Hameed et al.^42^ applied three ZnONPs levels to the basal diet of the broilers chicken (0, 40, and 110), where he estimated the best level in improving lactic acid bacteria count (1.2 × 107 = 7.08 log cfu) was 110 mg/kg compared to control diet (8.8 × 106 = 6.94 log cfu). Results suggested the use of Zn nanoparticles as alternative zinc source with less level, than usually applied, and with better results, which has a cleaner impact on the environment due to less incidence of zinc metal contamination^43^.

### Mode of action of nano zinc oxide in broilers

#### Productive performance

Nano zinc oxide (ZnONPs) improves broiler growth performance primarily through its superior bioavailability compared to conventional zinc sources^44^. Nanoparticles possess unique physicochemical properties that confer superior advantages over their conventional counterparts^2,10,45^. The nanoscale size allows for efficient intestinal absorption, reducing mineral losses and enhancing the utilization of zinc in metabolic processes. Moreover, ZnONPs readily participate in enzymatic reactions, thereby stimulating protein and energy metabolism and leading to improvements in feed conversion ratio (FCR)^46^. In addition, their potent antioxidative role is evident through activation of antioxidant enzymes such as superoxide dismutase (SOD) and glutathione peroxidase, which effectively minimize oxidative stress and support overall growth and health^47^.

#### Bone characteristics

The beneficial effects of ZnONPs extend to skeletal development, where they play a key role in improving bone mineralization and strength. By enhancing alkaline phosphatase activity, ZnONPs promotes calcium and phosphorus deposition in the bone matrix^48^. Furthermore, their nanoscale properties enable rapid penetration into bone cells, stimulating collagen formation and supporting the development of a denser and stronger skeletal structure. Consequently, broilers supplemented with ZnONPs exhibit accelerated bone growth and improved bone quality compared to those receiving conventional zinc sources^49^.

#### Gut microbiota

Nano-zinc exerts a significant modulatory effect on the intestinal microbiota, particularly through its antimicrobial activity against pathogenic bacteria such as Salmonella. ZnONPs can penetrate bacterial cell walls, disrupt membrane integrity, and cause leakage of cellular contents, ultimately leading to bacterial death^46,50^. Additionally, the release of Zn^2^⁺ ions interferes with bacterial enzymatic activity and biosynthetic pathways, inhibiting bacterial proliferation. Beyond its direct antimicrobial effect, ZnONPs enhances intestinal barrier integrity by strengthening tight junction proteins, thereby reducing the adhesion and translocation of Salmonella^51^. Importantly, ZnONPs selectively favor the proliferation of beneficial lactic acid bacteria (LAB) by suppressing pathogenic species such as E. coli and Salmonella. This selective effect, combined with increased production of short-chain fatty acids (SCFAs) and reduced intestinal pH, creates a favorable environment for LAB colonization^52^. Moreover, ZnONPs supports local mucosal immunity by enhancing immunoglobulin A (IgA) production, which facilitates LAB adhesion to the intestinal epithelium^53^.

In summary, ZnONPs supplementation in broilers offers multifaceted benefits, including enhanced growth performance, improved bone quality, and a healthier gut microbiota. Its superior bioavailability, strong antimicrobial properties against pathogens (particularly Salmonella), and supportive role in promoting beneficial bacteria distinguish it from conventional zinc sources, making it a promising feed additive for poultry production.

## Conclusion

According to our findings, the inclusion of nano zinc oxide provided the best productive performance; intestinal microbiota (Enhanced beneficial bacteria and reduced harmful bacteria). In addition to enhanced tibia bone characteristics by improved tibia weight, length, width and breaking strength also, enhanced bone mineral concentration and bone mineral density. As a result, the appropriate amount of nano zinc is 25%.

## Data Availability

The corresponding author can provide the datasets used in the current work upon reasonable request.
